# High-Performance Adaptive Weak Fault Diagnosis Based on the Global Parameter Optimization Model of a Cascaded Stochastic Resonance System

**DOI:** 10.3390/s23094429

**Published:** 2023-04-30

**Authors:** Zhihui Lai, Zhangjun Huang, Min Xu, Chen Wang, Junchen Xu, Cailiang Zhang, Ronghua Zhu, Zijian Qiao

**Affiliations:** 1 Shenzhen Key Laboratory of High Performance Nontraditional Manufacturing, College of Mechatronics and Control Engineering, Shenzhen University, Shenzhen 518060, China; laizh@szu.edu.cn (Z.L.);; 2 Guangdong Key Laboratory of Electromagnetic Control and Intelligent Robots, College of Mechatronics and Control Engineering, Shenzhen University, Shenzhen 518060, China; 3 Ningbo Cigarette Factory, China Tobacco Zhejiang Industry Co., Ltd., Ningbo 315040, China; 4 Ocean College, Zhejiang University, Zhoushan 316021, China; 5 School of Mechanical Engineering and Mechanics, Ningbo University, Ningbo 315211, China

**Keywords:** weak fault diagnosis, stochastic resonance, cascaded system, global parameter optimization, particle swarm optimization algorithm

## Abstract

Stochastic resonance (SR), as a type of noise-assisted signal processing method, has been widely applied in weak signal detection and mechanical weak fault diagnosis. In order to further improve the weak signal detection performance of SR-based approaches and realize high-performance weak fault diagnosis, a global parameter optimization (GPO) model of a cascaded SR system is proposed in this work. The cascaded SR systems, which involve multiple multi-parameter-adjusting SR systems with both bistable and tri-stable potential functions, are first introduced. The fixed-parameter optimization (FPO) model and the GPO models of the cascaded systems to achieve optimal SR outputs are proposed based on the particle swarm optimization (PSO) algorithm. Simulated results show that the GPO model is capable of achieving a better SR output compared to the FPO model with rather good robustness and stability in detecting low signal-to-noise ratio (SNR) weak signals, and the tri-stable cascaded SR system has a better weak signal detection performance compared to the bistable cascaded SR system. Furthermore, the weak fault diagnosis approach based on the GPO model of the tri-stable cascaded system is proposed, and two rolling bearing weak fault diagnosis experiments are performed, thus verifying the effectiveness of the proposed approach in high-performance adaptive weak fault diagnosis.

## 1. Introduction

Condition monitoring and fault diagnosis of rotating mechanical components, such as bearings, gears, and rotors, are important to ensure the safe operation of equipment [1]. Vibration analysis and magnetic flux leakage detection [2] are common and effective technologies in the condition monitoring and fault diagnosis of machinery. In many cases, especially when the machine operates in poor working conditions or the sensors have to be installed far away from the fault source, the fault characteristics are always submerged in strong background noise and other interference components, making them difficult to be identified. Therefore, many weak signal detection methods, such as spectral kurtosis (SK) [3], empirical mode decomposition (EMD) [4,5], digital filter [6], wavelet transform (WT) [7], etc., have been widely investigated to extract weak fault signals from noisy backgrounds. However, these weak signal detection methods inevitably weaken the fault characteristic when filtering out the noise; hence, the weak signal detection performance is limited. Different from the conventional weak signal detection methods, stochastic resonance (SR)-based methods can utilize the noise (rather than eliminate the noise) to achieve weak signal detection, thus showing potential in weak fault diagnosis.

The concept of SR was first proposed by Benzi et al. [8,9] in 1981 when they studied the paleo-meteorological problem of alternating glacial and warm climate cycles. When SR occurs, a part of the noise energy is transferred to the low-frequency signal through a nonlinear system. Thus, the low-frequency weak signal can be significantly enhanced while filtering out the high-frequency noise, and the output signal-to-noise ratio (SNR) is significantly improved [10,11]. Due to the advantages of SR in weak signal detection, SR has attracted continuous attention in the field of weak fault diagnosis in the past 40 years [12,13,14,15,16,17].

In order to make SR-based methods suitable for practical engineering applications of mechanical fault diagnosis, many efforts have been made by researchers. To overcome the limitation of conventional SR that it can only process a signal with small parameters (e.g., a signal frequency that is much less than 1 Hz) due to the limitation of the adiabatic approximation theory [18], researchers have proposed many solutions to make SR capable of processing large-parameter signals, such as frequency re-scaling SR (FRSR) [19], frequency-shifted and re-scaling SR (FSRSR) [20], parameter-normalized SR (PNSR) [21], modulated SR (MSR) [22], and scale-transformation SR [23], among others. To appropriately select the system parameters to generate the optimal SR output for given signals, many adaptive parameter-tuning SR methods have been investigated [10,18,24], which are further applied in the fault diagnosis of planetary gearboxes [24], rolling bearing fault diagnosis [25,26], and rotor misalignment fault diagnosis [27].

The most important objective of SR-based fault diagnosis methods is to achieve a high-performance weak signal detection ability. The weak signal detection ability of an SR-based method is related to the potential function of the system [12]. It was reported that SR can be produced in nonlinear systems with monostable, bistable, tri-stable, and multi-stable potential functions [23,28,29,30,31]. Moreover, many novel forms of potential functions, such as the Woods–Saxon potential function proposed by Gao et al. [32], the pining potential function employed by Zhang et al. [33], the piecewise linear potential function employed by Li et al. [34], and the compound tri-stable potential function proposed by Tang et al. [1], have been proposed recently for the realization of SR and the successful application of weak signal detection. However, how to quantitively and reasonably evaluate the detection performance of an SR system with different potential functions is still an issue that needs to be solved.

Moreover, the weak signal detection ability of a single SR system is still limited [35], especially for weak signals with extremely low SNRs. Hence, some enhancement models based on the SR systems, such as the cascaded SR systems [36,37,38], coupled SR systems [29,39], and parallel SR systems [40,41], have been constructed to further enhance the weak signal detection ability of SR-based methods. Among these enhancement models, the cascaded SR models, which consist of several single SR systems connected in series, are of most interest. It is known that a single SR system acts as a low-pass filter that can transfer the energy from a high-frequency region to a low-frequency region, thus amplifying the low-frequency weak signal [42]. Hence, multiple filtering processes can be realized through a cascaded SR system, thus making the output signal smoother and making the characteristic signal more obvious. Therefore, the cascaded SR system may have a better weak signal detection performance compared to a single SR system.

Compared to a single SR system, the number of adjustable parameters of a cascaded system is significantly enhanced, and different parameter-adjusting strategies have been proposed for a cascaded system to realize SR output. Up to now, the so-called “fixed-parameter adjusting strategy” and “stage-by-stage parameter adjusting strategy” have been the most adopted. The fixed-parameter strategy means that the corresponding parameters in each subsystem are kept the same. Using this parameter-adjusting strategy, Hairan et al. [43] proposed a signal detection method under a dual-mode non-Gaussian noise background based on a cascaded SR. This parameter-adjusting strategy can be further combined with a multi-parameter adaptive optimization algorithm to find out the optimal SR output adaptively [44]. The stage-by-stage parameter-adjusting strategy means the optimal SR output of each subsystem is obtained by adjusting the parameters of this subsystem, and its SR output is input into the next subsystem (if necessary). Therefore, the parameters of each subsystem may be different. Using this strategy, Xiao et al. [45] proposed an adaptive cascaded vibration resonance method and applied it to rotating machinery fault diagnosis; Liu et al. [46] proposed an adaptive cascaded piecewise linear SR system for bearing fault diagnosis; Xu et al. [37] proposed an adaptive cascaded SR method using the quantum particle swarm optimization (QPSO) algorithm; and Zhang et al. [47] proposed a cascaded three-steady-state SR and applied it to rolling bearing fault diagnosis. However, it is noted that in most of the literature, this strategy is adopted for a cascaded system with only two subsystems, as the output SNR cannot continue to increase with more subsystems. Hence, the fixed-parameter-adjusting strategy can produce a better SR output compared to the stage-by-stage parameter-adjusting strategy. Furthermore, it can be easily understood that as the cascaded system is not treated as a system as a whole, the optimal SR output of the cascaded system cannot be found by either the “fixed-parameter adjusting strategy” or the “stage-by-stage parameter adjusting strategy”. In contrast, a novel “global-parameter adjusting strategy”, which means all parameters of all subsystems are adjusted jointly, has the potential to realize the optimal SR output for the whole cascaded system. This parameter-adjusting strategy, which has not been fully studied yet, is worthy to be further investigated.

Therefore, in this paper, the global parameter optimization model of a cascaded SR system is proposed for high-performance adaptive weak signal detection and weak fault diagnosis. As it can achieve the theoretically optimal SR output of a cascaded system, the output SNR of this model can be also used to quantitively evaluate the detection performance of SR systems with different potential functions. In this paper, the classical bistable and tri-stable potential functions are considered for demonstration. The rest of the paper is organized as follows. In Section 2, the background knowledge of the multi-parameter-adjusting SR is introduced, and both the fixed-parameter optimization (FPO) model and the global parameter optimization (GPO) model of cascaded SR systems are constructed with the help of the particle swarm optimization (PSO) algorithm. The weak signal detection performance of the single SR system and the FPO model and the GPO model of the cascaded systems with either bistable or tri-stable potential functions is fully studied and compared through numerical simulations in Section 3. Two rolling bearing weak fault diagnosis experiments are performed in Section 4 to demonstrate the effectiveness of the proposed GPO model of a cascaded system in weak signal detection and weak fault diagnosis. Conclusions are drawn in Section 5.

## 2. Weak Signal Detection Approaches Based on Adaptive Cascaded SR Systems

### 2.1. Multi-Parameter-Adjusting SR

SR is a nonlinear physical phenomenon reflecting the synergistic effect of the nonlinear system, the weak periodic signal, and the noise. When SR occurs, the weak periodic signal is enhanced by gaining energy from high-frequency noise. The mathematical model of an underdamped SR system, which describes the motion of a unit-mass Brownian particle in a potential field in the presence of periodic force and noise, can be written as:

(1) $$\frac{dx^{2}\left(t\right)}{dt^{2}}+k\frac{dx\left(t\right)}{dt}=-\frac{dU\left(x\right)}{dx}+s(t)+n(t)$$

Here, k denotes the damping ratio; U(x) denotes the potential field of the system, and −dU(x)/dx is the potential field force applied to the particle; $s(t)=A\cos(2\pi f_{0}t)$ represents a harmonic signal with amplitude A and frequency $f_{0}$; and $n(t)=\sqrt{2D}\xi (t)$ represents noise with an intensity of D, where ξ(t) is zero-mean and unit-variance Gaussian white noise. In this system, sn(t)=s(t)+n(t) is defined as the input signal, and x(t) denotes the output signal, which can be obtained by numerically solving the equation.

The nonlinear system shown in Equation (1) can generate SR with many different potential fields. Among these, the bistable potential field and the tri-stable potential field are two classical potential fields, whose generalized potential functions can be written as follows [28,48]:

(2) $$U_{B}\left(x\right)=-\frac{1}{2}a_{1}x^{2}+\frac{1}{4}b_{1}x^{4}$$

(3) $$U_{T}\left(x\right)=\frac{1}{2}a_{2}x^{2}-\frac{1}{4}b_{2}x^{4}+\frac{1}{6}c_{2}x^{6}$$

where the potential field parameters $a_{1}$, $b_{1}$, $a_{2}$, $b_{2}$, and $c_{2}$ all have positive values.

**Figure 1:**
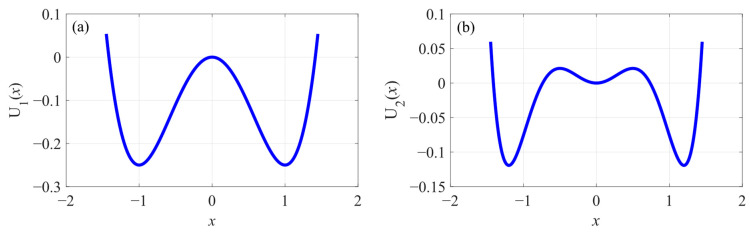
Potential functions of (**a**) the bistable potential field with $a_{1}=1$ and $b_{1}=1$ and (**b**) the tri-stable potential field with $a_{2}=0.36$, $b_{2}=1.69$, and $c_{2}=1.69$.

Examples of these two potential functions are plotted in Figure 1. It can be seen that the bistable potential field is symmetrical with two potential wells, and the tri-stable one is symmetrical with three potential wells.

The output signal x(t) of the SR system can be understood as the displacement of the Brownian particle moving in the potential field driven by the periodic signal and the random noise. When the signal is weak and noise is absent, i.e., $A<A_{C}$ and D=0 ($A_{C}$ indicates the critical amplitude for the particle to pass over the potential barrier to another potential well [48]), the Brownian particle can only oscillate within one of the potential wells. When noise is present (D>0), the particle may pass over the barrier even though the signal is weak, as the particle can accumulate energy from high-frequency noise with the help of the nonlinear system. When the transition rate of the particle between potential wells accords with the signal period, the particle can realize regular inter-well oscillation, and SR occurs. Hence, the weak features of the input signal can be enhanced through SR and can be identified from the output signal x(t). This advantage makes SR an advanced weak signal detection model, which has been applied in many fields including weak fault diagnosis.

Due to the limitation of the adiabatic approximation theory [23], the classical SR system shown in Equation (1) is only applicable to small-parameter conditions ($A<A_{C}$, $f_{0}\ll 1$, and D≪1), which limits its applications in practical engineering. To solve this issue, a multi-parameter-adjusting SR system is further proposed by introducing an amplitude-transformation coefficient ε and a scale-transformation coefficient R to Equation (1), which can be written as [23]:

(4) $$\frac{dx^{2}\left(t^{\prime }\right)}{d{t^{\prime }}^{2}}+k\frac{dx\left(t^{\prime }\right)}{dt^{\prime }}=-\frac{dU\left(x\right)}{dx}+\varepsilon \cdot sn(t^{\prime })$$

Here, ε is used to linearly transform the amplitude of the input signal to be in an appropriate small-parameter range, and $t^{\prime }=Rt$ is the times series after scale transformation, where R is used to transform the frequency/time scale of the input signal to meet the small-parameter conditions. The scale transformation can be simply realized by adopting a time step of $h^{\prime }=R/F_{s}$ instead of $h=1/F_{s}$ in the calculation when numerically solving the mathematic models of the SR systems [23], where $F_{s}$ is the sampling frequency of the input signal. Thus, the frequency of the characteristic signal can be regarded as $f_{0}{}^{\prime }=f_{0}/R$ in the calculations, and the small-frequency condition of $f_{0}{}^{\prime }\ll 1$ can be realized by appropriately adjusting the value of R. Hence, SR can be achieved under large-parameter conditions using the multi-parameter-adjusting SR system.

### 2.2. Models of Cascaded SR Systems

**Figure 2:**
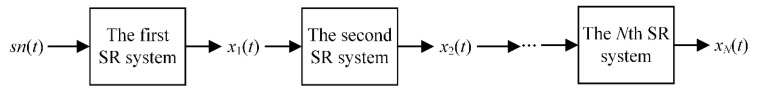
Framework of a cascaded SR system.

The SR system has shown its potential in weak signal detection, but its detection ability for a weak signal with an extremely low SNR is still limited, which should be further improved. One possible solution is to construct a cascaded SR system, which represents multiple SR systems connected in series, as shown in Figure 2. Note that the number of connected subsystems is defined as N in this work. Previous research shows that as the low-frequency weak signal can continuously accumulate energy from high-frequency signal and noise through multiple SR systems, the cascaded SR system has better output performance compared to the single SR system, such as a higher output SNR, a more obvious signal spectral peak, and a smoother output signal resulting from multiple high-frequency filterings [35].

In order to utilize the cascade SR system to detect a large-parameter weak signal, the multi-parameters (k, ε, R, and the potential field parameters) of each subsystem should be optimized to achieve an optimal SR output. For this purpose, the multi-parameters can be optimized through different optimization models of the cascaded SR system. The first one is termed the fixed-parameter optimization (FPO) model of the cascade SR system, as shown in Equation (5), which has been studied by many scholars previously [44,49]. One can see that the multi-parameters in all subsystems stay the same. Thus, the cascaded bistable SR system only has five parameters (k, $a_{1}$, $b_{1}$, ε, and R) to be optimized, and the cascaded tri-stable SR system only has six parameters (k, $a_{2}$, $b_{2}$, $c_{2}$, ε, and R) to be optimized. In the optimization process, the objective of the FPO model is to realize the optimal SR output of the last SR system by optimizing these limited parameters. Therefore, the optimization process based on the FPO model is relatively simple due to the small quantity of the parameters to be optimized.

(5) $$\left\{ \begin{array}{l} \frac{dx_{1}{}^{2}\left(t^{\prime }\right)}{d{t^{\prime }}^{2}}+k\frac{dx_{1}\left(t^{\prime }\right)}{dt^{\prime }}=-\frac{dU\left(x_{1}\right)}{dx_{1}}+\varepsilon \cdot sn(t^{\prime })\\ \frac{dx_{2}{}^{2}\left(t^{\prime }\right)}{d{t^{\prime }}^{2}}+k\frac{dx_{2}\left(t^{\prime }\right)}{dt^{\prime }}=-\frac{dU\left(x_{2}\right)}{dx_{2}}+x_{1}(t^{\prime })\\ \ldots \\ \frac{dx_{N}{}^{2}\left(t^{\prime }\right)}{d{t^{\prime }}^{2}}+k\frac{dx_{N}\left(t^{\prime }\right)}{dt^{\prime }}=-\frac{dU\left(x_{N}\right)}{dx_{N}}+x_{N-1}(t^{\prime }) \end{array}\right.$$

In order to further improve the SR output of the cascaded SR system, a novel optimization model of the cascaded SR system is proposed in this work, which is termed the global parameter optimization (GPO) model, as shown in Equation (6). It can be seen that in this model, the multi-parameters of each subsystem are independent. In the optimization process, the objective is to realize the optimal SR output of the last subsystem by optimizing all multi-parameters jointly, and the number of parameters to be optimized is decided by the number of connected subsystems (N). It is easy to understand that the optimized result obtained from the FPO model is one of the special cases of the results obtained from the GPO model. Therefore, the proposed GPO model has the potential to achieve a better SR output compared to the FPO model.

(6) $$\left\{ \begin{array}{l} \frac{dx_{1}{}^{2}\left(t^{\prime }\right)}{d{t^{\prime }}^{2}}+k_{1}\frac{dx_{1}\left(t^{\prime }\right)}{dt^{\prime }}=-\frac{dU_{1}\left(x_{1}\right)}{dx_{1}}+\varepsilon \cdot sn(t^{\prime })\\ \frac{dx_{2}{}^{2}\left(t^{\prime }\right)}{d{t^{\prime }}^{2}}+k_{2}\frac{dx_{2}\left(t^{\prime }\right)}{dt^{\prime }}=-\frac{dU_{2}\left(x_{2}\right)}{dx_{2}}+x_{1}(t^{\prime })\\ \ldots \\ \frac{dx_{N}{}^{2}\left(t^{\prime }\right)}{d{t^{\prime }}^{2}}+k_{N}\frac{dx_{N}\left(t^{\prime }\right)}{dt^{\prime }}=-\frac{dU_{N}\left(x_{N}\right)}{dx_{N}}+x_{N-1}(t^{\prime }) \end{array}\right.$$

### 2.3. Optimization of the Cascaded SR System Based on the PSO Algorithm

To achieve the optimal SR output of both the FPO model and the GPO model of the cascaded system containing many adjustable parameters, all these parameters should be optimized through a multi-parameter optimization algorithm, which should be carefully selected to match the optimization objective. The particle swarm optimization (PSO) algorithm is a global algorithm based on the foraging behavior of birds. This method requires fewer parameters to adjust compared with other algorithms such as the genetic algorithm and the mosquito swarm, making it easier to implement and simplify the algorithm [28]. Therefore, the PSO algorithm is adopted in this work to find out the optimal SR output of both the FPO model and the GPO model of the cascaded system. The optimization process is briefly introduced in this subsection.

In order to evaluate the output performance of the cascaded SR system appropriately, the output SNR (${\mathrm{SNR}}_{\mathrm{out}}$) of the system is first defined as the evaluation index [28]:

(7) $${\mathrm{SNR}}_{\mathrm{out}}=10\log_{10}\left(\frac{P_{0}{}^{2}}{{\sum \left[X\left(f\right)\right]}^{2}-P_{0}{}^{2}}\right)$$

where X(f) represents the single-side spectrum of the output signal of the single or cascade SR system and $P_{0}$ is the amplitude value of the output signal at the characteristic frequency. It is assumed that the optimal SR output of the single or cascaded system is achieved when ${\mathrm{SNR}}_{\mathrm{out}}$ reaches its maximum value. Hence, ${\mathrm{SNR}}_{\mathrm{out}}$ can be also used as the objective function of the PSO algorithm.

The PSO algorithm can be described as follows: assuming a particle swarm is a population of n particles in a Z-dimensional search space, The position and velocity of the ith particle are defined as $X_{i}={\left(x_{i1},\;x_{i2},\;x_{i3},\;\ldots \;,\;x_{iZ}\right)}^{T}$ and $V_{i}={\left(V_{i1},\;V_{i2},\;V_{i3},\;\ldots \;,\;V_{iZ}\right)}^{T}$, respectively. Initially, random position and velocity populations are created. In each iteration, the particles update their own by tracking two optimal solutions: one is the individual optimal solution of each particle $p_{i}=\left(p_{i1},\;p_{i2},\;p_{i3},\;\ldots \;,\;p_{iZ}\right)$, and the other one is the population optimal solution of all particles $p_{g}=\left(p_{g1},\;p_{g2},\;p_{g3},\;\ldots \;,\;p_{gZ}\right)$ [10]. Then, the positions and velocities are updated according to the iterative formulas; thus, the individual and population optimal solution can be updated accordingly at each iteration. The optimization process is finished when the number of iterations reaches the pre-set value $I_{\max}$, and the final population extreme value and the corresponding position of the particle can be obtained. In optimizing the cascaded system, the final population extreme value indicates the optimal ${\mathrm{SNR}}_{\mathrm{out}}$ of the system, and the optimal parameter indicates the population optimal solution. Moreover, the iterative formulas of the velocity and position are given as:

(8) $$V_{i,\;j}\left(t+1\right)=\omega V_{i,\;j}\left(t\right)+c_{1}r_{1}\left[p_{i,\;j}-x_{i,\;j}\left(t\right)\right]+c_{2}r_{2}\left[p_{g,\;j}-x_{i,\;j}\left(t\right)\right]$$

(9) $$x_{i,\;j}\left(t+1\right)=x_{i,\;j}\left(t\right)+V_{i,\;j}\left(t+1\right),\;j=1,\;\ldots \;,\;Z$$

where $c_{1}$ and $c_{2}$ are positive learning factors or acceleration constants and $r_{1}$ and $r_{2}$ are random numbers uniformly distributed within 0~1. In order to effectively control the flight velocity of the particle, an inertia weight ω is also introduced. In addition, by setting the velocity range $\left[V_{\min},\;V_{\max}\right]$ and position range $\left[x_{\min},\;x_{\max}\right]$ of the particle, the movement of the particle can be appropriately limited. In this paper, $c_{1}$ and $c_{2}$ are set to 1.718, and ω is set to 0.8.

In this paper, the optimal ${\mathrm{SNR}}_{\mathrm{out}}$ of the signal SR system and the FPO model and the GPO model of a cascaded SR system are obtained by using the PSO algorithm. The only difference in the optimization of these three systems is their optimized parameters.

## 3. Numerical Simulations

### 3.1. Weak Signal Detection Based on the FPO Model and the GPO Model

In this subsection, the weak signal detection results of the FPO model and GPO model of the cascaded bistable system are studied and compared by processing a simulated weak signal. The cascaded tri-stable SR system has similar results, which are not presented in this paper. It is noted that the fourth-order Runge–Kutta algorithm is adopted in this work to solve the equations of the SR systems.

**Figure 3:**
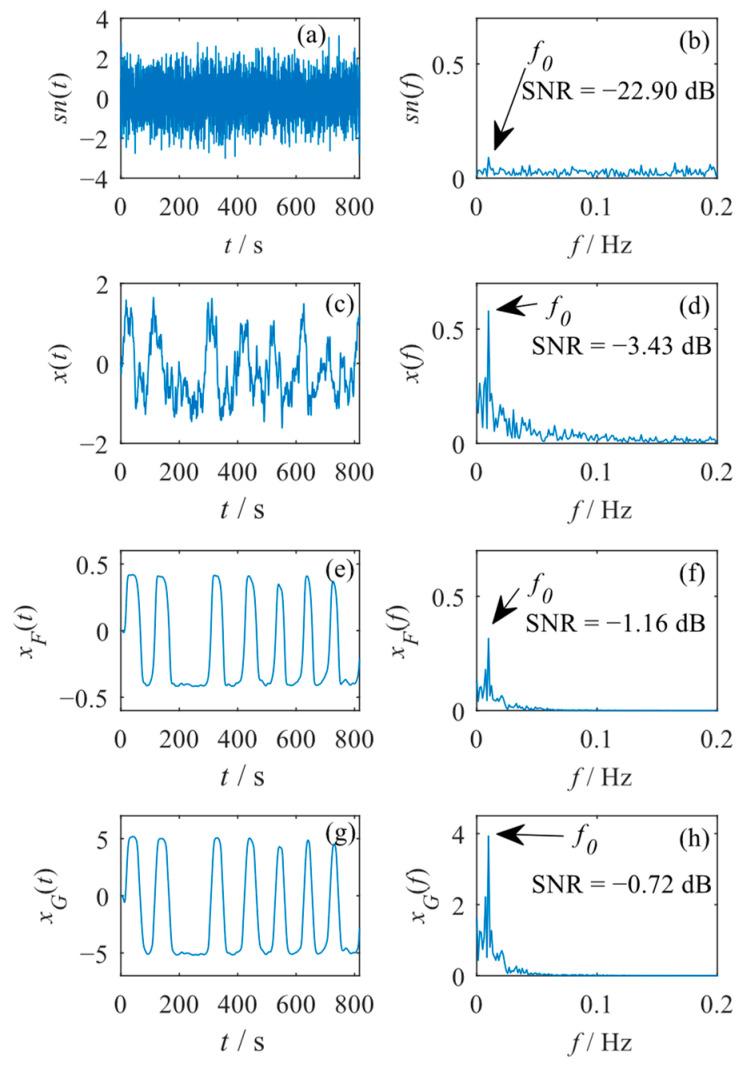
The waveform and spectrum of (**a**,**b**) the weak signal, (**c**,**d**) the output signal of the single bistable system, (**e**,**f**) the output signal of the FPO model of the cascaded bistable system (N=4), and (**g**,**h**) the output signal of the GPO model of the cascaded bistable system (N=4).

A weak signal sn(t) with A=0.1, $f_{0}=0.01$ Hz, and D=0.4 is first generated. The sampling frequency of the signal is set to $F_{s}=5$ Hz, and the signal contains 4096 points. The waveform sn(t) and its spectrum sn(f) are shown in Figure 3a,b. One can see that the characteristic signal is almost submerged in the strong background noise due to a relatively low ${\mathrm{SNR}}_{\mathrm{in}}$ of −22.90 dB, which can be calculated similarly to Equation (7). This weak signal is further processed by the single SR system Equation (4), the FPO model Equation (5), and the GPO model Equation (6) of a cascaded SR system (N=4) with the bistable potential function Equation (2). It is noted that in the optimization processes, the values of ε and R are both set to 1, and the optimization ranges of other parameters are all set to [0.01, 6]. Moreover, the number of iterations $I_{\max}$ is set to 200, and the population n is set to 30 in the PSO algorithm.

First, the weak signal sn(t) is processed by the single bistable SR system. In the optimization process, the values of k, $a_{1}$, and $b_{1}$ are optimized to achieve an optimal SR output. The optimization result shows that with sn(t) input, the single bistable system achieves optimal SR when $\left[k,\;a_{1},\;b_{1}]=[1.58,\;0.08,\;0.20\right]$, and the optimal ${\mathrm{SNR}}_{\mathrm{out}}$ is −3.43 dB. The output waveform x(t) and its spectrum x(f) are shown in Figure 3c,d. It can be seen that the characteristic of the weak signal is significantly enhanced after being processed by the single bistable system.

Next, the FPO model of the cascaded bistable system (N=4) is used to process the weak signal sn(t). The optimized parameters contain k, $a_{1}$, and $b_{1}$. An optimal ${\mathrm{SNR}}_{\mathrm{out}}$ of −1.16 dB can be obtained from the FPO model when $\left[k,\;a_{1},\;b_{1}]=[5.24,\;0.01,\;5.67\right]$. The output waveform $x_{F}\left(t\right)$ of the FPO model and its spectrum $x_{F}\left(f\right)$ are shown in Figure 3e,f. One can see that the characteristic of the weak signal is further enhanced through the FPO model by filtering other interference signals.

Lastly, the weak signal sn(t) is processed by the GPO model of the cascaded bistable system (N=4). In this case, there exist 12 optimized parameters including $k_{1}$, $a_{11}$, $b_{11}$, $k_{2}$, $a_{12}$, $b_{12}$, …, $k_{4}$, $a_{14}$, and $b_{14}$. When their values are 0.54, 0.01, 0.01, 4.74, 0.01, 0.03, 5.91, 0.95, 3.06, 2.25, 0.03, and 0.01, respectively, the GPO model achieves its optimal ${\mathrm{SNR}}_{\mathrm{out}}$ of −0.72 dB. The output waveform $x_{G}\left(t\right)$ of the GPO model and its spectrum $x_{G}\left(f\right)$ are shown in Figure 3g,h. It can be seen that the spectral peak at the frequency of $f_{0}=0.01$ Hz is more obvious compared to previous results.

The simulated results show that by improving the signal spectral peak and filtering the interference signals/noise, the characteristic of the weak signal can be significantly enhanced through the single bistable system and the FPO model and the GPO model of the cascaded bistable system. The FPO model and the GPO model can achieve better SR outputs compared to the single bistable system, demonstrating the priority of the cascaded SR system in weak signal detection. Moreover, the GPO model is capable of achieving a better SR output compared to the FPO model, which accords with our previous analysis in Section 3.1. However, these conclusions are obtained from a special simulation case, which should be further demonstrated through statistical analysis. Moreover, it should be noted that the single bistable system and the FPO model and the GPO model of the cascaded bistable system all can detect the frequencies of multiple frequency components by setting the corresponding detected frequencies, and they will not misidentify non-existent frequency components. This can be demonstrated through further simulations, which are not presented in this paper.

### 3.2. Statistical Analysis of the Weak Signal Detection Performances of Different Models

In this subsection, the weak signal detection performances of different models are fully investigated through statistical analysis. In this paper, six models are studied, including the single SR model, the FPO model, and the GPO model of the cascaded SR system with both bistable and tri-stable potential functions, as shown in Table 1.

In the simulations, a weak signal with A=0.1 and $f_{0}=0.01$ Hz is generated, and noises with intensities of 0.4, 1.6, 2.8, and 4.0 are added. Thus, four weak signals with an ${\mathrm{SNR}}_{\mathrm{in}}$ of −22.90 dB, −28.65 dB, −30.61 dB, and −31.66 dB are obtained. The noisy signals are processed by the presented 6 models to obtain their optimal SR results, and the searching ranges of the optimization parameters are all set to [0.01, 6] except ε=1 and R=1. Every signal is processed by each model 100 times, and the results are analyzed and compared statistically.

**Figure 4:**
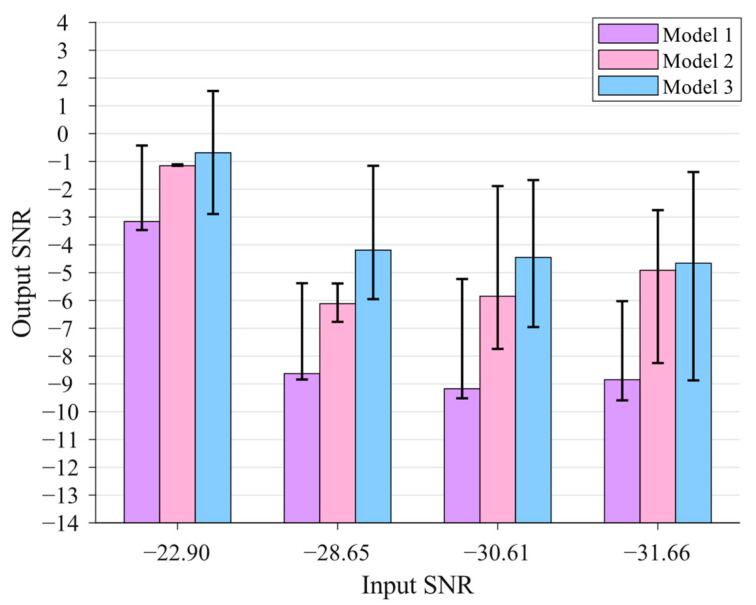
The statistical graph of the optimization results based on Models 1–3.

The statistical graph of the optimization results based on Models 1–3 is shown in Figure 4, in which the mean value of the output SNRs of each model for each input signal is plotted using a histogram and the maximal and minimum ${\mathrm{SNR}}_{\mathrm{out}}$ for each case are plotted as well. One can see from Figure 4 that all the ${\mathrm{SNR}}_{\mathrm{out}}$ are much larger than their corresponding ${\mathrm{SNR}}_{\mathrm{in}}$, indicating that all presented SR systems can significantly enhance the characteristic of the weak signal using the PSO algorithm. Moreover, for each input signal, the highest average ${\mathrm{SNR}}_{\mathrm{out}}$ can be achieved from Model 3, and the second highest average ${\mathrm{SNR}}_{\mathrm{out}}$ can be achieved from Model 2, indicating that in general, the GPO model of the cascaded bistable system has the best weak signal detection performance compared to the FPO model of the cascaded bistable system (second best) and the single bistable system. This conclusion can be also drawn from the fact that Model 3 can produce the highest maximum ${\mathrm{SNR}}_{\mathrm{out}}$ for each input signal and also the highest minimum ${\mathrm{SNR}}_{\mathrm{out}}$ in most cases. Another interesting finding is that as the ${\mathrm{SNR}}_{\mathrm{in}}$ decreases from −28.65 dB to −31.66 dB, the average ${\mathrm{SNR}}_{\mathrm{out}}$ for each case maintains a relatively stable value, demonstrating the robustness and stability of the SR systems in detecting low-SNR weak signals.

**Figure 5:**
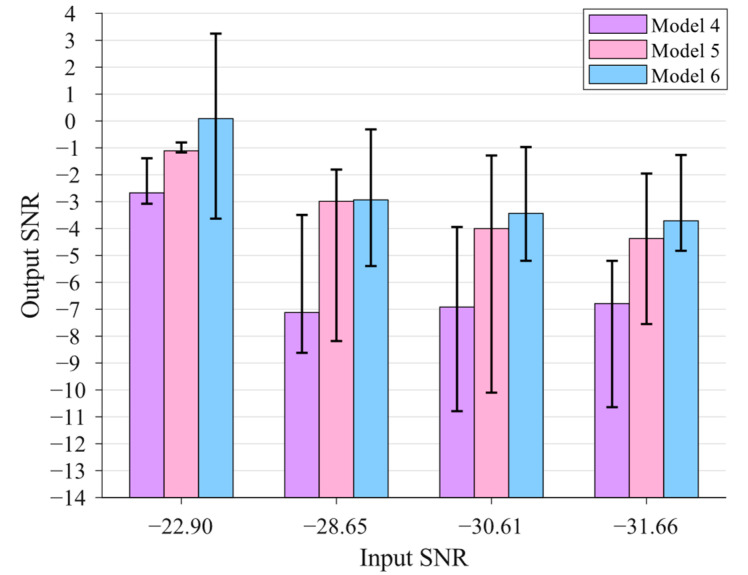
The statistical graph of the optimization results based on Models 4–6.

The statistical graph of the optimization results based on Models 4–6 is further shown in Figure 5. It can be seen that the statistical graph shows a similar pattern to Figure 4, indicating that the conclusions of the weak signal detection performance based on the bistable systems are applicable to those based on the tri-stable systems.

**Figure 6:**
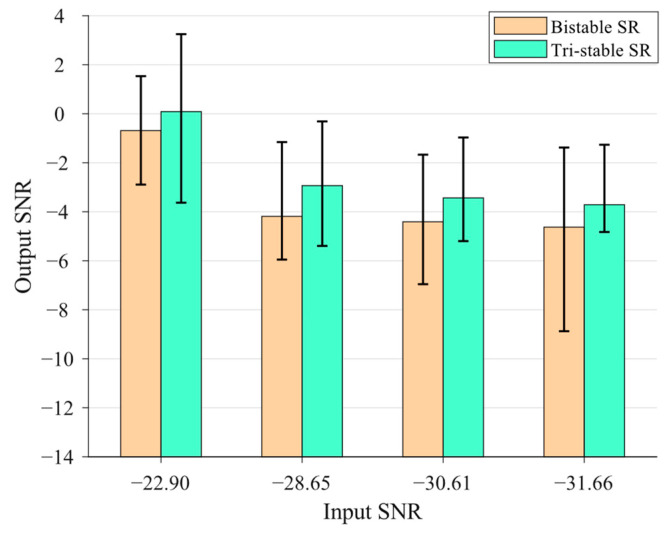
The statistical graph of the optimization results based on Model 3 and Model 6.

Previous analyses show the superiority of the proposed GPO model of the cascaded SR system compared to the conventional FPO model of the cascaded SR system and the single SR system. Furthermore, the weak signal detection performance of the cascaded systems with a bistable potential function and tri-stable potential function is compared through statistical analysis. In Figure 6, the mean values, maximum values, and minimum values of the output SNRs in 100 simulations based on the GPO models of the cascaded bistable systems and tri-stable systems are plotted for comparison. It can be seen that in most cases with different weak signals, especially the low-${\mathrm{SNR}}_{\mathrm{in}}$ cases, the GPO model of the cascaded tri-stable system has a higher average ${\mathrm{SNR}}_{\mathrm{out}}$, higher maximum ${\mathrm{SNR}}_{\mathrm{out}}$, and higher minimum ${\mathrm{SNR}}_{\mathrm{out}}$ (except for one case) compared to those of the cascaded bistable system. This result indicates that the tri-stable SR system has a better weak signal detection performance compared to the bistable SR system, which has been reported in Refs. [28,50] for single SR systems.

### 3.3. Influence of the Number of Subsystems

In previous simulations, the cascaded SR systems were pre-set as four-stage cascaded systems. Actually, the number of subsystems (N) has an influence on the weak signal detection performance and optimization time of the cascaded system, which are fully investigated in this subsection.

**Figure 7:**
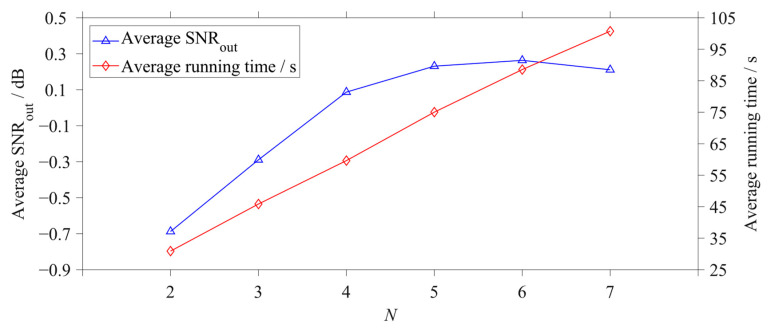
The statistical graph of the optimization results based on Model 6 with different numbers of subsystems.

The GPO model of the cascaded tri-stable system is used for investigation. A total of 5 cascaded tri-stable systems with N=2, 3, 4, 5, 6, 7, are adopted to analyze the same noisy signal with A=0.1, $f_{0}=0.01$ Hz, and D=0.4. The searching ranges of all adjustable parameters are set to [0.01, 6] except ε=1 and R=1; the number of iterations $I_{\max}$ is set to 200, and the population n is set to 30 in the PSO algorithm. The input signal is processed by each cascaded system 100 times, and the average ${\mathrm{SNR}}_{\mathrm{out}}$ and running times of each case are presented in Figure 7.

It can be seen from Figure 7 that as N increases from 2 to 4, the average ${\mathrm{SNR}}_{\mathrm{out}}$ gradually increases, which stays almost constant when the number of subsystems continues to increase from 4 to 6. The average ${\mathrm{SNR}}_{\mathrm{out}}$ value starts to decrease when N increases to 7. The reason is that when the number of subsystems is large, too many subsystem parameters make it easy for the whole system to achieve a local optimum instead of the global optimum. On the other hand, with N increasing, the number of optimized parameters increases accordingly, and the average running time increases linearly, which can be seen in Figure 7. Therefore, the four-stage cascaded system is a high-cost-performance cascaded system that has a relatively high output performance and an acceptable optimization time. In the following sections of this paper, four-stage cascaded systems are used for weak signal detection and fault diagnosis.

## 4. Experimental Verifications

**Figure 8:**
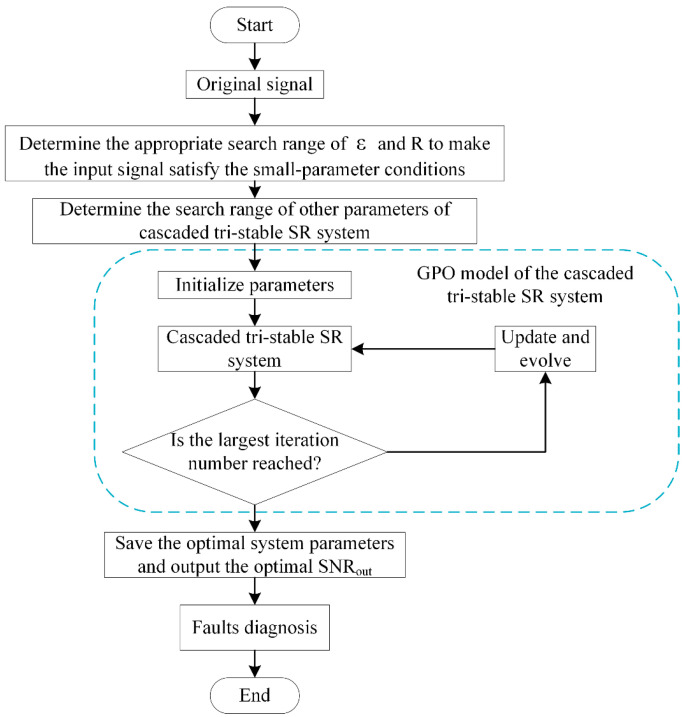
Flowchart of the fault diagnosis based on the GPO model of the cascaded tri-stable system.

The simulation results of Section 3 show that the GPO model of the cascaded SR system has a rather good weak signal detection performance. Hence, it has great potential in mechanical weak fault diagnosis for faulty signals with extremely low SNR. In this section, two examples of practical mechanical weak fault diagnosis are presented to verify the effectiveness of the proposed GPO model of the cascaded SR system. It is noted that the SR system with tri-stable potential functions is used due to its better weak signal detection performance compared to that with a bistable potential function. The fault diagnosis process is shown in Figure 8.

### 4.1. Case 1: Diagnosis of Rolling Bearing Inner Raceway Fault

The data of the rolling bearing inner ring fault are obtained from the bearing data center website of the Case Western Reserve University (CWRU). The associated test rig consists of a 2-horsepower reliance electric motor, a torque transducer/encoder, a dynamometer, and control electronics. In the tests, a groove of width of 0.021 inches was cut in the face of the inner raceway of the drive-end bearing by using electrical discharge machining (EDM), thus introducing a single-point inner ring fault. The faulty bearing was a deep groove ball bearing (6205-2RS JEM SKF), whose parameters are shown in Table 2. According to the fault mechanism of the inner raceway fault, a characteristic frequency of $f_{i}$ is contained in the vibration signal of the vibrating system. The value of $f_{i}$ can be calculated according to [51]:

(10) $$f_{i}=\frac{z}{2}\left(1+\frac{d_{r}}{D_{p}}\cos\beta \right)f_{R}$$

where z is the number of rolling elements; $d_{r}$ and $D_{p}$ are the rolling elements’ diameter and the pitch diameter, respectively; β is the contact angle; and $f_{R}$ is the rotation frequency of the bearing. During the test, the shaft speed was 1774 rpm, i.e., $f_{R}=29.57$ Hz; thus, the characteristic frequency of the inner raceway fault can be calculated as $f_{i}=160.1$ Hz.

**Figure 9:**
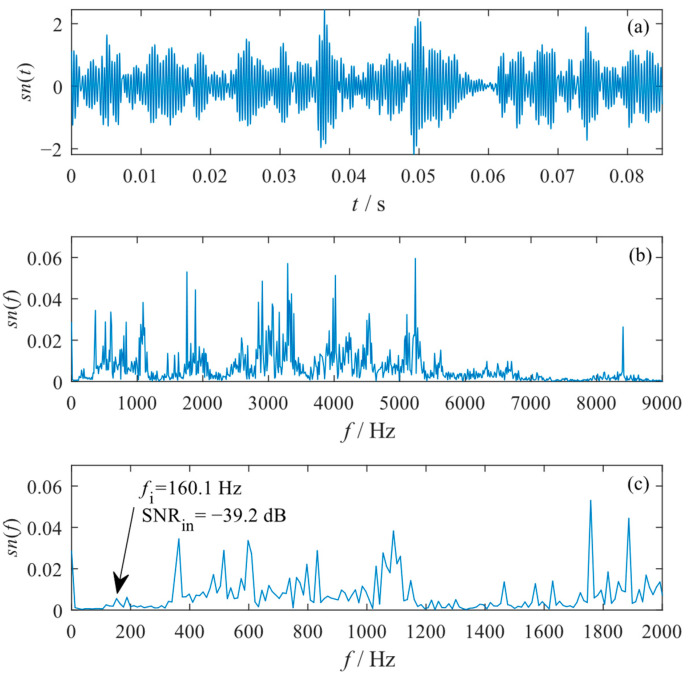
Original vibration signal with bearing inner raceway fault. (**a**) Waveform; (**b**) global spectrum; (**c**) low-frequency spectrum.

The bearing vibration signals were acquired using an accelerometer placed at the motor housing, and the sampling frequency was 48,000 Hz. The length of the original signal used in this paper was M=4096, and its input SNR was calculated as ${\mathrm{SNR}}_{\mathrm{in}}=-39.45$ dB. The waveform, global spectrum, and low-frequency spectrum of the original signal are shown in Figure 9. It can be seen that the fault characteristic frequency of $f_{i}=160.1$ Hz cannot be identified from either the time domain or the frequency domain due to the strong noise interference. Therefore, the existing inner raceway fault of the bearing cannot be diagnosed directly from the original signal.

**Figure 10:**
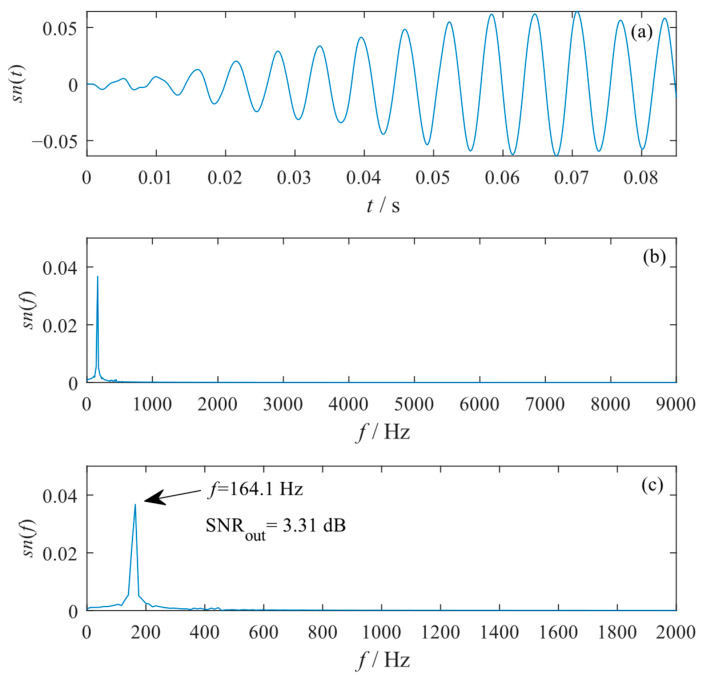
The output signal obtained from the 4-stage cascaded tri-stable system optimized by the GPO model with the rolling bearing inner raceway faulty signal input. (**a**) Waveform; (**b**) global spectrum; (**c**) low-frequency spectrum.

In order to diagnose the potential inner raceway fault of the rolling bearing, the proposed GPO model of a four-stage cascaded tri-stable system was adopted to further analyze the original signal. In the optimization of the PSO algorithm, the ${\mathrm{SNR}}_{\mathrm{out}}$ at the frequency closest to the characteristic frequency $f_{i}$ was selected as the objective function. The searching ranges of the amplitude-transformation coefficient and the scale-transformation coefficient were set to ε∈[0,30] and R∈[2000,3000], respectively, and the searching ranges of other optimization parameters ($a_{1},b_{1},c_{1},k_{1},a_{2},b_{2},c_{2},k_{2},\ldots ,a_{4},b_{4},c_{4},k_{4}$) were all set to [0.01,6]. The values of the optimal parameters of ε, R, $a_{1}$, $b_{1}$, $c_{1}$, $k_{1}$, $a_{2}$, $b_{2}$, $c_{2}$, $k_{2}$, … , $a_{4}$, $b_{4}$, $c_{4}$, and $k_{4}$ obtained from the GPO model were 6.13, 2000.60, 5.95, 1.10, 1.07, 4.48, 5.39, 4.63, 2.77, 3.72, 3.79, 5.08, 0.93, 4.77, 0.27, 2.38, 1.62, and 0.01, respectively, which enabled the cascaded tri-stable system to produce an optimal SR output with an optimal ${\mathrm{SNR}}_{\mathrm{out}}$ of +3.31 dB. The corresponding waveform, global spectrum, and low-frequency spectrum of the output signal were presented in Figure 10. One can see from the waveform that the system output presents a regular inter-well oscillation, and a spectral peak is prominent at f=164.1 Hz with other interference components greatly reduced and nearly eliminated. It can be calculated that the frequency resolution of the spectrum is $\Delta f=F_{s}/N=11.7$ Hz; thus, the frequency of the prominent spectral peak (f=164.1 Hz) indicates the characteristic frequency of an inner raceway fault ($f_{i}=160.1$ Hz). Hence, an inner raceway fault in the experimental bearing can be identified from the system SR output, which accords with the practical truth. Thus, the effectiveness of the proposed GPO model of the cascaded SR system in mechanical fault diagnosis can be verified.

### 4.2. Case 2: Diagnosis of Rolling Bearing Outer Raceway Fault

**Figure 11:**
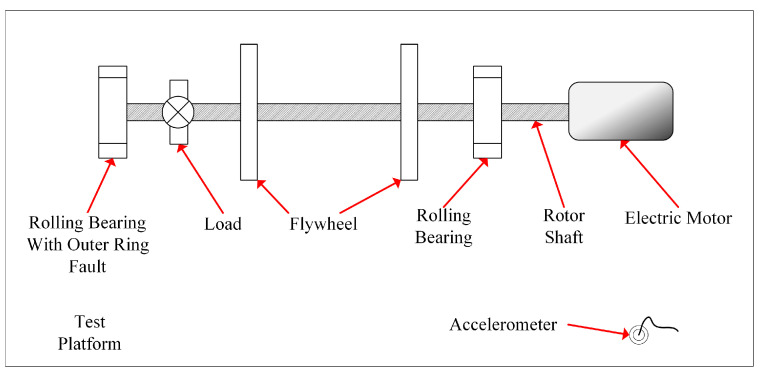
Bearing outer raceway fault test rig.

The bearing outer raceway failure test rig is shown in Figure 11, which mainly contains a rotor shaft and two rolling bearings driven by an electric motor. In the tests, the outer raceway of the left bearing (cylindrical roller bearing, NU205) had a groove 0.2 mm in width and 0.1 mm in depth, which simulated a bearing with an outer raceway fault. According to the fault mechanism of the outer raceway fault, a characteristic frequency of $f_{o}$ is contained in the vibration signal of the vibrating system, whose value can be calculated according to [51]:

(11) $$f_{o}=\frac{z}{2}\left(1-\frac{d_{r}}{D_{p}}\cos\beta \right)f_{R}$$

In the tests, the motor rotated at a speed of 950 rpm, i.e., $f_{R}=15.83$ Hz. Hence, $f_{o}=82.95$ Hz can be obtained with basic parameters of the faulty bearing of z=13, β=0°, $\;d_{r}=7.5\;$ mm, and $D_{p}=39\;$ mm.

**Figure 12:**
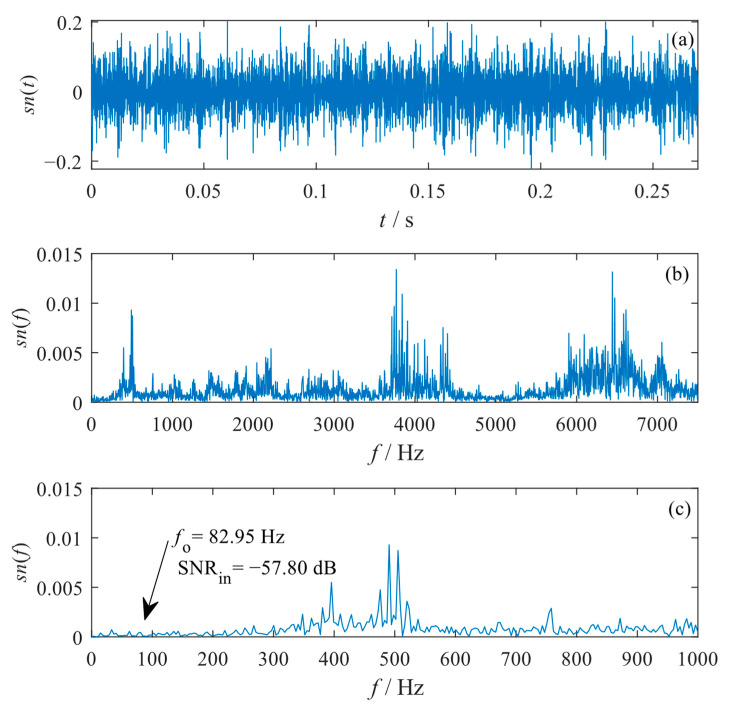
Original vibration signal with bearing outer raceway fault. (**a**) Waveform; (**b**) global spectrum; (**c**) low-frequency spectrum.

An accelerometer was installed on the test rig to acquire the vibration signal of the system using a signal-acquiring device (NI PXI-1033). In the tests, the sampling frequency was $F_{s}=$ 15,000 Hz. An original signal with length M=4096 was used for analysis. The waveform, global spectrum, and low-frequency spectrum of the original signal are shown in Figure 12. It can be seen that as the SNR of the original signal was as low as ${\mathrm{SNR}}_{\mathrm{in}}=-39.45$ dB, the waveform is disordered, and the fault characteristic is also submerged in the spectrum. Therefore, the outer raceway fault of the bearing cannot be identified from the original signal.

**Figure 13:**
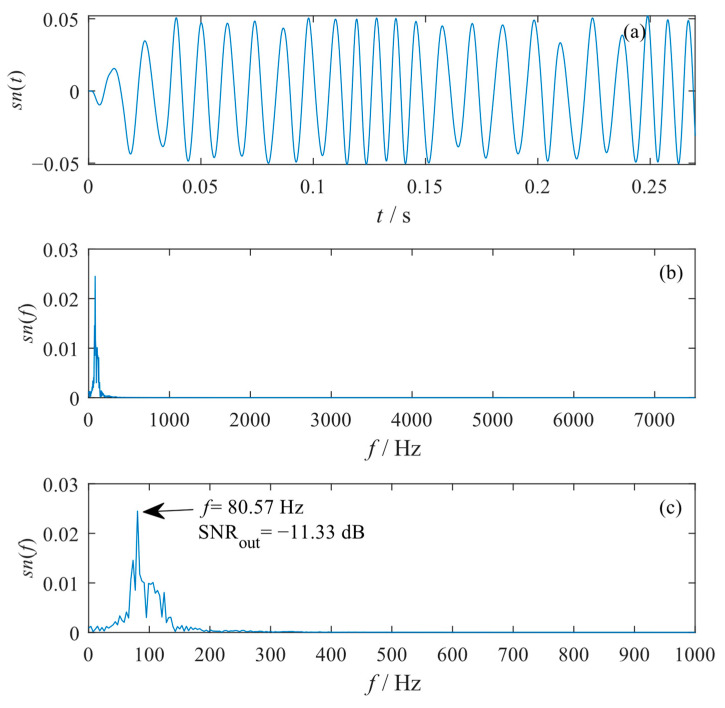
The output signal obtained from the 4-stage cascaded tri-stable system optimized by the GPO model with the rolling bearing outer raceway faulty signal input. (**a**) Waveform; (**b**) global spectrum; (**c**) low-frequency spectrum.

Again, the proposed GPO model of the four-stage cascaded tri-stable system was used to further process the original signal. The searching ranges of the amplitude-transformation coefficient and the scale-transformation coefficient were set to ε∈[0,100] and R∈[1000,2000], respectively, and the searching ranges of $a_{1},\;b_{1},\;c_{1},\;k_{1},\;a_{2},\;b_{2},\;c_{2},\;k_{2},\;\ldots \;,\;a_{4},\;b_{4},\;c_{4},\;k_{4}$ were all set to [0.01,6]. The optimal SR output (${\mathrm{SNR}}_{\mathrm{out}}=-11.33$ dB) was achieved when the values of ε, R, $a_{1}$, $b_{1}$, $c_{1}$, $k_{1}$, $a_{2}$, $b_{2}$, $c_{2}$, $k_{2}$, … , $a_{4}$, $b_{4}$, $c_{4}$, and $k_{4}$ were 74.06, 1002.90, 3.40, 2.81, 3.48, 3.88, 0.27, 0.97, 2.41, 0.01, 4.19, 3.44, 2.59, 3.49, 3.00, 5.16, 1.56, and 3.90, respectively. The corresponding waveform and spectrum of the output signal are presented in Figure 13. It can be seen from the waveform that the output signal presents a regular inter-well oscillation. The most prominent component has a frequency of 80.57 Hz, which can be clearly observed from the spectrum. Considering the frequency resolution of $\Delta f=F_{s}/N=3.66$ Hz, this characteristic frequency indicates an outer raceway fault frequency of 82.95 Hz. Thus, an outer raceway fault in the rolling bearing can be identified, and the effectiveness of the proposed methods in mechanical fault diagnosis is verified again.

## 5. Conclusions

In this paper, by combining the multi-parameter-adjusting cascaded stochastic resonance (SR) system and the particle swarm optimization (PSO) algorithm, a global parameter optimization (GPO) model of a cascaded SR system is proposed for high-performance adaptive weak fault diagnosis. The cascaded SR system may involve different potential functions and contain multiple SR systems connected in series. Simulated results show that the GPO model of a cascaded SR system can realize adaptive weak signal detection even under large-parameter conditions with a better performance compared to the single SR system and the pre-proposed fixed-parameter optimization (FPO) model of the cascaded SR system, and the robustness and stability of the system in detecting low-SNR weak signals are rather good. Moreover, the cascaded SR system with a tri-stable potential function has a higher probability of producing a better SR output compared to that with a bistable potential function. The influence of the number of subsystems on the system output is also investigated. It is found that a four-stage cascaded system is a high-cost-performance cascaded system that has a relatively high output performance and also an acceptable optimization time. The weak signal detection and weak fault diagnosis approach is further proposed based on the GPO model of a tri-stable cascaded SR system, which is applied to diagnose the inner raceway fault and the outer raceway fault of a rolling bearing. Experimental work shows that the proposed approach can be utilized to identify the weak characteristic frequency component submerged in a noisy background by significantly enhancing the signal-to-noise ratio (SNR) of the characteristic signal, thus demonstrating its effectiveness in high-performance adaptive mechanical weak fault diagnosis.

## Figures and Tables

**Table 1:** The SR models investigated in this work.

	Bistable Potential Field	Tri-Stable Potential Field
Single SR system	Model 1	Model 4
FPO model of the cascaded SR system	Model 2	Model 5
GPO model of the cascaded SR system	Model 3	Model 6

**Table 2:** The parameters of the tested rolling bearing.

Model of Bearing	Inside Diameter $d_{r}$ (Inches)	Outside Diameter (Inches)	Thickness (Inches)	Ball Diameter (Inches)	No. of Rolling Elements *Z*	Contact Angle β
SKF 6205-2RS JEM	0.9843	2.0472	0.5906	0.3126	9	0°

## Data Availability

Data are available on request from the authors.

## Abbreviations

The following abbreviations are used in this manuscript:

CWRU	Case Western Reserve University
EDM	Electrical Discharge Machining
EMD	Empirical Mode Decomposition
FPO	Fixed-parameter Optimization
FRSR	Frequency Re-scaling Stochastic Resonance
FSRSR	Frequency-Shifted and Re-scaling Stochastic Resonance
GPO	Global parameter Optimization
MSR	Modulated Stochastic Resonance
PNSR	Parameter-Normalized Stochastic Resonance
PSO	Particle Swarm Optimization
QPSO	Quantum Particle Swarm Optimization
SK	Spectral Kurtosis
SNR	Signal-to-noise Ration
SR	Stochastic Resonance
WT	Wavelet Transform
